# Report of a new species of sand fly, *Phlebotomus (Anaphlebotomus) ajithii* n. sp. (Diptera: Psychodidae), from Western Ghats, India

**DOI:** 10.1186/s13071-024-06468-2

**Published:** 2024-09-12

**Authors:** Harish Kumar Shah, P. A. Fathima, Jose Jicksy, Prasanta Saini

**Affiliations:** ICMR-Vector Control Research Centre, Field Station, Kottayam, Kerala India

**Keywords:** *Phlebotomus (Anaphlebotomus) ajithii*, Phlebotomine sand flies, *COI* barcode, Western Ghats, India

## Abstract

**Background:**

Western Ghats is a biodiversity treasure trove with reports of indigenous leishmaniasis cases. Hence, systematic sand fly surveillance was carried out among the tribal population. The present study reports a novel sand fly species, *Phlebotomus (Anaphlebotomus) ajithii* n. sp. (Diptera: Psychodidae), discovered in the Western Ghats of India.

**Methods:**

A comprehensive sand fly survey was conducted across the Kollam, Thrissur, Idukki, Kasaragod and Malappuram districts of Kerala, India. The survey spanned both indoor and outdoor habitats using standard collection methods over a 3-year, 3-month period. DNA barcoding of samples was performed targeting mitochondrial cytochrome *c* oxidase subunit I (*COI*) gene, and the sequence generated was subjected to phylogenetic analysis.

**Results:**

*Phlebotomus (Anaphlebotomus) ajithii*, a new sand fly species, is recorded and described in this communication. The morphological relationship of the new species to other members of the subgenus *Anaphlebotomus* is discussed. Mitochondrial *COI* barcode followed by phylogenetic analysis confirmed that specimens of *Ph. ajithii* belong to the same taxonomic group, while a genetic distance of 11.7% from congeners established it as a distinct species.

**Conclusions:**

The Western Ghats, known for its rich biodiversity, has lacked systematic entomological surveys focusing on sand flies. This study aims to fill this gap and reports and describes a new species of sand fly.

**Graphical abstract:**

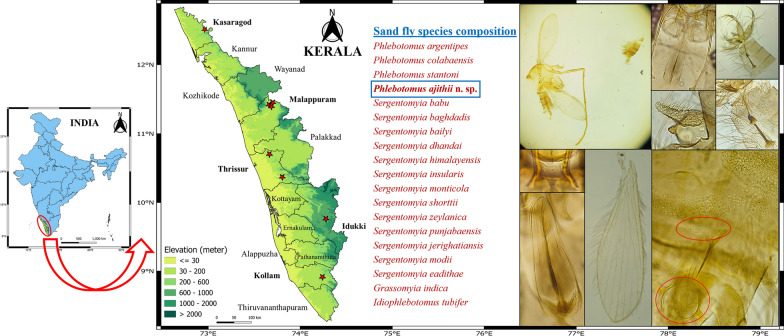

## Background

Phlebotomine sand flies are classified within the Psychodidae family under the order Diptera, and they are diminutive, hairy insects that feed on blood. These insects are of immense public health importance since they play a role as vectors of leishmaniasis. Leishmaniasis, a neglected tropical disease (NTD), is caused by a flagellate protozoan parasite from the *Leishmania* genus. It has been endemic in many countries like India, Brazil, Sudan, Bangladesh, Ethiopia and Nepal for centuries [1].

The Western Ghats, also known as the Sahyadri, is a biological treasure trove that boasts a rich biodiversity comprising numerous endemic species of plants, fishes, amphibians, reptiles and mammals [2, 3]. In India, the Western Ghats expands from Gujarat to Tamil Nadu with an area of 160,000 km^2^ and is considered one of 34 biodiversity hotspots worldwide [4]. Several tribal populations belonging to different ethnic groups are inhabitants of this zone. Human inhabitation and the practice of agriculture have made several alterations in the ecology and landscape of the Western Ghats [5]. In addition, this region is also relevant in terms of different vector-borne diseases, transmitted by insects, ticks, mites, etc. [6–8].

Western Ghats is emerging as an endemic belt for visceral leishmaniasis (VL) and cutaneous leishmaniasis (CL) in the last few decades, with many new indigenous case records [9]. Over the last 2 decades, nearly 50 cases of CL and VL were reported from the tribal population of these zones [10, 11]. Sand flies are the only proven vector of the *Leishmania* parasite involved in the transmission of leishmaniasis. The availability of resting and breeding habitats and the many blood meals via various hosts have aided in the abundant survival of sand flies in this region [12]. From 1990 till 2022, many new species and country records of sand flies have been documented from the Western Ghats through various entomological survey carried out by a few researchers [13–22]. Later, all these species were combined and included in a review article with a total of the 69 sand fly species recorded in India; almost 50% those species are found in Kerala [23]. Hence, considering the rich biodiversity and endemicity of the disease, systematic sand fly surveillance has been carried out across the Western Ghats of Kerala, especially in the tribal belts. A new species of sand fly was identified during this survey, *Phlebotomus (Anaphlebotomus) ajithii* n. sp. Taxonomic and phylogenetic characterizations of the species are documented in this article.

## Methods

### Study area

**Fig. 1:**
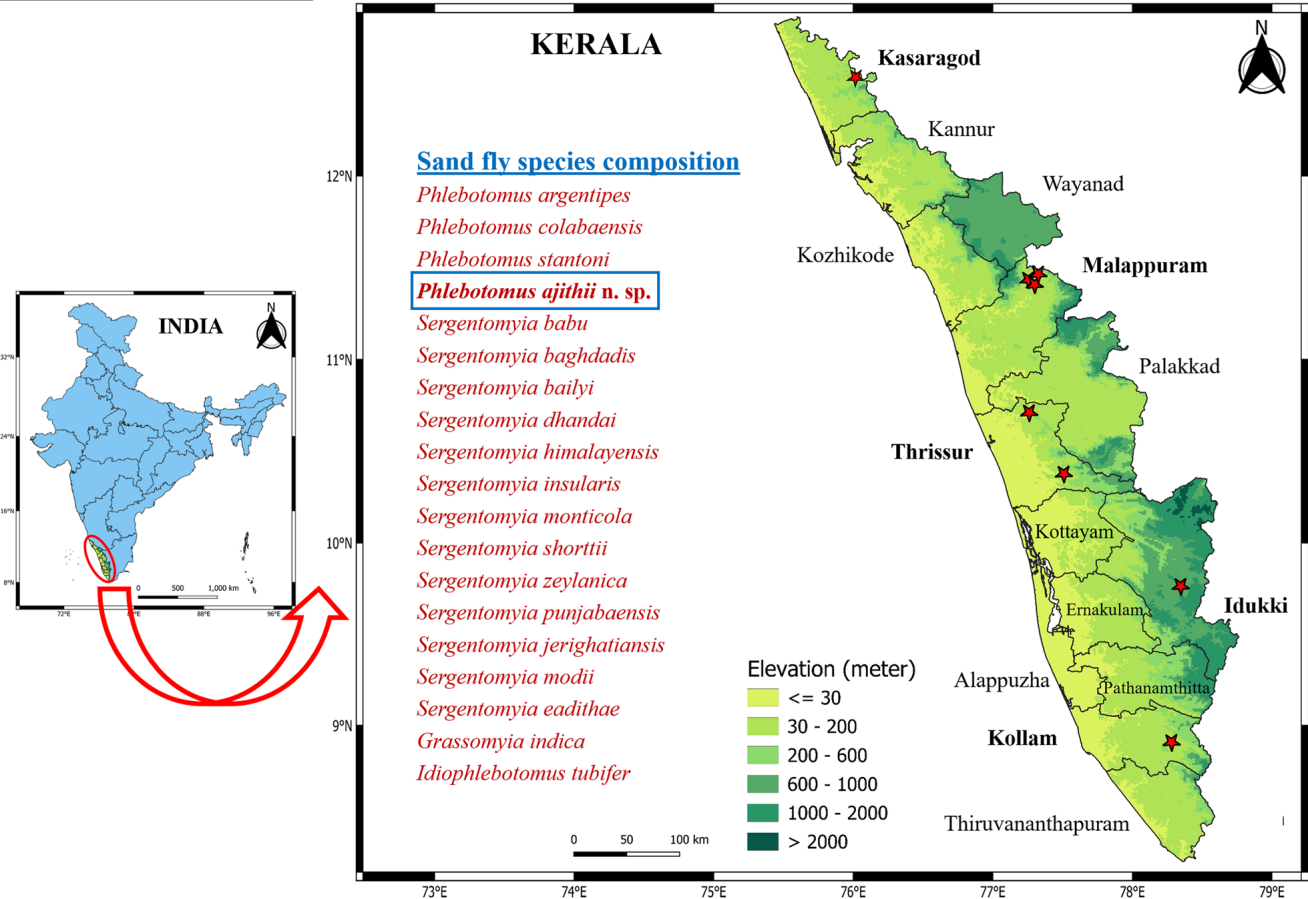
Sand fly specimen collection area in Thiruvananthapuram, Kollam, Thrissur, Idukki, Kasaragod and Malappuram districts of Kerala, India, along with species composition

The entomological investigation was conducted within the various tribal communities situated in Western Ghats of Kollam, Thrissur, Idukki, Kasaragod and Malappuram districts in Kerala from January 2021–March 2024 (Table 1 and Fig. 1).

**Table 1:** Details of entomological survey study area

Sample no.	State	District	Panchayat	Taluk	Settlement (tribe)	GPS coordinates
1	Kerala	Thrissur	Mattathoor	Mukundapuram	Sasthampoovam (Kadaar)	10.36 N, 76.44 E
2	Kerala	Thrissur	Mullurkkara	Thalapilly	Mullurkara (Kadaar)	10.69 N, 76.25 E
3	Kerala	Kollam	Kulathupuzha	Pathanapuram	Cheukara (Kani)	8.93 N, 77.03 E
4	Kerala	Idukki	Kanchiyar	Idukki	Anjuruli (Malayarayar, Mannan, Ulladan)	9.77 N, 77.08 E
5	Kerala	Kasaragod	Panathadi	Vellarikund	Ottamala (Maratti, Maavilan)	12.48 N, 75.33 E
5	Kerala	Malappuram	Pothukallu	Nilambur	Pothukallu (Paniyan, Kattunaikkan)	11.40 N, 76.25 E
6	Kerala	Malappuram	Chungathara	Nilambur	Chungathara (Paniyan, Malappanikkan, Kurumar, Muthuvan)	11.37 N, 76.28 E
7	Kerala	Malappuram	Edakkara	Nilambur	Edakkara (Paniyan, Aranadan, Malappanikkan)	11.43 N, 76.30 E

These tribal settlements are scattered throughout the mountainous forest expanse of the Western Ghats in southern India. Referred to locally as the Sahyadri, this elevated area in Kerala lies on the periphery of the Deccan Plateau, demarcating it from the coastline strip along the Arabian Ocean. The Ghats boasts diverse vegetation, encompassing grasslands, arid and humid temperate forests, evergreen and semi-evergreen woodlands, scrub jungles and more. Coconut, rubber, pepper, jackfruit and teak plantations within these forests serve as major sources of livelihood for the indigenous tribes. The rich cultural heritage of the region is safeguarded by its intricate topography and abundant rainfall. Moreover, the forests within these ranges are designated as reserve forests and are patrolled by forest rangers appointed by the Kerala Government.

The tribes residing within these mountain ranges now inhabit dwellings constructed of concrete blocks, a measure introduced by the Kerala Government as part of an initiative aimed at the development of the tribal population. Reliant on the forest for their livelihood, these tribes often migrate to the remote reaches of the forest. Consequently, the abandoned houses, left vacant amid the moisture and humidity brought by the rains, create an ideal microhabitat for the breeding of sand flies. Within these settlements, houses are spaced roughly 100–200 m apart, totaling around 50–100 dwellings. Sand fly collection was conducted using standard entomological collection methods such as mechanical aspirators, light and sticky traps both indoors (cattle sheds and human dwellings) and outdoors (rodent burrows, tree holes, termite mounts etc.). On the other hand, resting collections employing mechanical aspirators were carried out in the dawn hours, between 9:00 a.m. and 12:00 noon. Trap collections, on the other hand, were undertaken from 6:00 p.m. (the preceding day) to 6:00 a.m. (the following day), covering the evening and night hours.

### Morphological identification

Sand fly specimens were brought to Indian Council of Medical Research-Vector Control Research Centre (ICMR-VCRC), Field Station at Kottayam, and preserved in 70% ethanol. The samples were dissected under stereomicroscope (Weswox Optik-SZM-100, India) and mounted in Hoyer’s medium on microscopic slides. Identification at species level was carried out by examining specimens under binocular microscope (Primostar 3, Carl Zeiss Suzhou Co., Ltd., China) with reference to standard taxonomic keys (Table 2) [13, 24, 25]. Some specimens (female and male) did not match the characters of the reported species in the keys and published literature. Taxonomic features of these specimens are distinct from the congeners sand fly species (subgenus *Anaphlebotomus*) such as *Phlebotomus (Ana.) colabaensis* Young and Chalam, 1927*, Ph. (Ana.) stantoni* Newstead, 1914, and *Ph. (Ana.) hoepplii* Tang and Maa, 1945, which have been previously explained [13]. The morphometric analysis of the possible new sand fly species was carried out using a Zeiss binocular microscope aided with a micrometer. Every measurement was logged in micrometers (µm). Images of unique identifying features of these specimens were taken using a camera mounted over the same compound microscope. A holotype female and allotype male along with nine paratype female samples were used for morphometric analysis. Terminologies of the attributes for description were adopted from Galati et al. [26]. Nomenclature was adopted following the guidelines given by the International Code of Zoological Nomenclature (ICZN) [27]. Measurements (µm) of *Ph. (Ana.) ajithii* n. sp. (holotype female and allotype male) are described below (Table 3).

**Table 2:** Species composition of phlebotomine sand flies collected and identified in the present study

Genus	Species	Females	Males	Total
*Phlebotomus*	*argentipes*	459	323	782
	*colabaensis*	46	12	58
	*stantoni*	26	8	34
	*ajithii* n. sp.	54	15	69
*Sergentomyia*	*babu*	60	50	110
	*baghdadis*	19	10	29
	*dhandai*	14	4	18
	*himalayensis*	32	18	50
	*insularis*	23	8	31
	*monticola*	29	16	45
	*shorttii*	8	0	8
	*zeylanica*	58	23	81
	*punjabaensis*	6	3	9
	*jerighatiansis*	49	24	73
	*modii*	1	1	2
	*eadithae*	2	0	2
*Grassomyia*	*indica*	2	0	2
*Idiophlebotomus*	*tubifer*	2	1	3
Total		890	516	1406

**Table 3:** Morphometric parameters of female and male *Phlebotomus (Anaphlebotomus) ajithii* n. sp. (in µm)

Morphometric parameters	Female (*N* = 10)				Male (*N* = 10)			
	Max	Min	Mean	SD	Max	Min	Mean	SD
Body length	1950	1890	1920	13	1775	1700	1733	24
Head length	460	420	442	13	390	370	384	7
Head width	400	360	386	13	370	360	364	5
Interocular distance	180	150	163	11	150	147	149	1
Clypeus	175	158	168	8	150	148	149	1
Labarum	280	250	268	11	185	175	183	4
No. of teeth on hypopharynx	–	–	18	–	–	–	–	–
No. of maxillary ventral teeth	–	–	17	–	–	–	–	–
No. of maxillary lateral teeth	–	–	10	–	–	–	–	–
Palpomere length P1	140	120	128	7	100	95	98	3
Palpomere length P2	165	145	155	7	109	106	108	2
Palpomere length P3	165	150	155	6	110	105	108	3
Palpomere length P4	85	70	75	5	70	65	68	3
Palpomere length P5	190	175	180	6	165	155	161	4
Antenna I (A I )	275	250	255	7	235	225	230	5
Antenna II (A II)	110	100	106	4	110	100	103	3
Antenna III (A III)	110	100	106	4	110	100	103	3
Ascoid in A II	68	65	66	1	53	50	51	1
No. of teeth in cibarium	Cibarium with few horizontal teeth				Cibarium with no visible teeth			
Pharynx length	193	185	189	3	160	155	158	2
Pharynx width	78	70	74	3	45	43	44	1
Pharyngeal armature (depth/length)	68	60	63	3	43	43	43	0
Wing length	1805	1725	1786	23	1500	1450	1478	18
Wing width	600	525	567	33	500	450	477	18
Principal vein length								
Alpha	440	410	430	13	300	300	300	0
Beta	300	270	283	14	230	220	226	5
Gamma	250	210	232	17	110	110	110	0
Delta	110	90	102	7	50	50	50	0
R5 length	1300	1225	1252	27	940	910	927	11
Foreleg								
Coxa	325	250	275	24	275	275	275	0
Trochanter	100	75	79	10	75	75	75	0
Femur	800	750	763	17	600	575	593	12
Tibia (T)	1075	975	1013	27	850	825	835	13
Tarsomeres								
T1	675	625	642	22	575	525	550	20
T2	275	250	263	13	250	250	250	0
T3	200	175	188	13	150	150	150	0
T4	175	150	163	13	125	125	125	0
T5	100	100	100	0	100	100	100	0
Length of spermatheca	40	38	39	1	–	–	–	–
Width of spermatheca	18	15	16	1	–	–	–	–
Number of segmentations in spermathecal	15	13	–	–	–	–	–	–
Length of common spermathecal duct	60	48	54	4	–	–	–	–
Length of spermathecal duct	585–780 (individual duct is highly coiled)				–	–	–	–
Length of cerci	150	143	146	3	–	–	–	–
Genital furca	68	60	63	3	–	–	–	–
Length of sperm pump	–	–	–	–	205	200	204	2
Length of aedeagal duct	–	–	–	–	180	170	175	4
Length of sperm pump + length of aedeagal duct	–	–	–	–	385	370	378	5
Ratio of length of sperm pump/length of aedeagal duct	–	–	–	–	1.21	1.14	1.17	0.03
Length of paramere	–	–	–	–	150	145	148	3
Length of ejaculatory apodeme	–	–	–	–	155	150	152	3
Length of epandrial lobes	–	–	–	–	230	220	226	4
Gonocoxite length	–	–	–	–	205	200	203	3
Gonostyle length	–	–	–	–	150	150	150	0
Gonostyle spine length	–	–	–	–	110	105	108	3

*N* number of specimens, *Max* maximum, *Min* minimum, *SD* standard deviation, *R *radius, ‘–’ not applicable

### Molecular identification

The genomic DNA was extracted from both the legs and entire body of individual sand flies using the QIAmp DNeasy Kit (Qiagen, Germany), adhering to the manufacturer's instructions. The sand fly specimen was homogeneously crushed with a mortar and pestle, and final DNA elution was done in 30 μl molecular-grade nuclease-free water. DNA barcoding of both female and male samples was performed targeting mitochondrial cytochrome *c* oxidase subunit I (*COI*) gene (~ 720 bp). The gene was amplified according to the procedure outlined by Kumar et al. [28]. Sequencing of the amplicons was done bi-directionally using the same set of primers. The nucleotide sequences generated were deposited in GenBank.

### Phylogenetic analyses

For phylogenetic analysis, the sequences were blasted with nucleotide repository (GenBank), and the sequences most similar to resulting sequences were aligned using MEGA 7.0. The sequences aligned with other congeners were analysed for phylogenetic tree construction using neighbor-joining statistical method with Kimura 2.0 parameter and 10,000 bootstraps. The genetic distance and other related parameters were estimated using MEGA 7.0.

### Natural infection assessment of *Leishmania* parasite

Since the possible new species of sand fly belongs to the *Phlebotomus* genus, assessment for natural infection of *Leishmania* parasite was carried out. The whole genomic DNA was subjected to real-time detection of *Leishmania* kinetoplast minicircle DNA (kDNA). The protocol for kDNA detection was followed as described by Castelli et al. [29] using primers LEISH-1 (5ʹ GGCGTTCTGCGAAAACCG 3ʹ), LEISH-2 (5ʹ AAAATGGCATTTTCGGGCC 3ʹ) and TaqMan probe (5ʹ FAM-TGGGTGCAGAAATCCCGTTCA 3ʹ-BHQ1).

## Results

Family Psychodidae Newman, 1834.

Subfamily Phlebotominae Rondani & Berté, in Rondani 1840.

Genus *Phlebotomus* Rondani & Berté, in Rondani 1840.

Subgenus *Anaphlebotomus* Theodor, 1948.

**Fig. 2:**
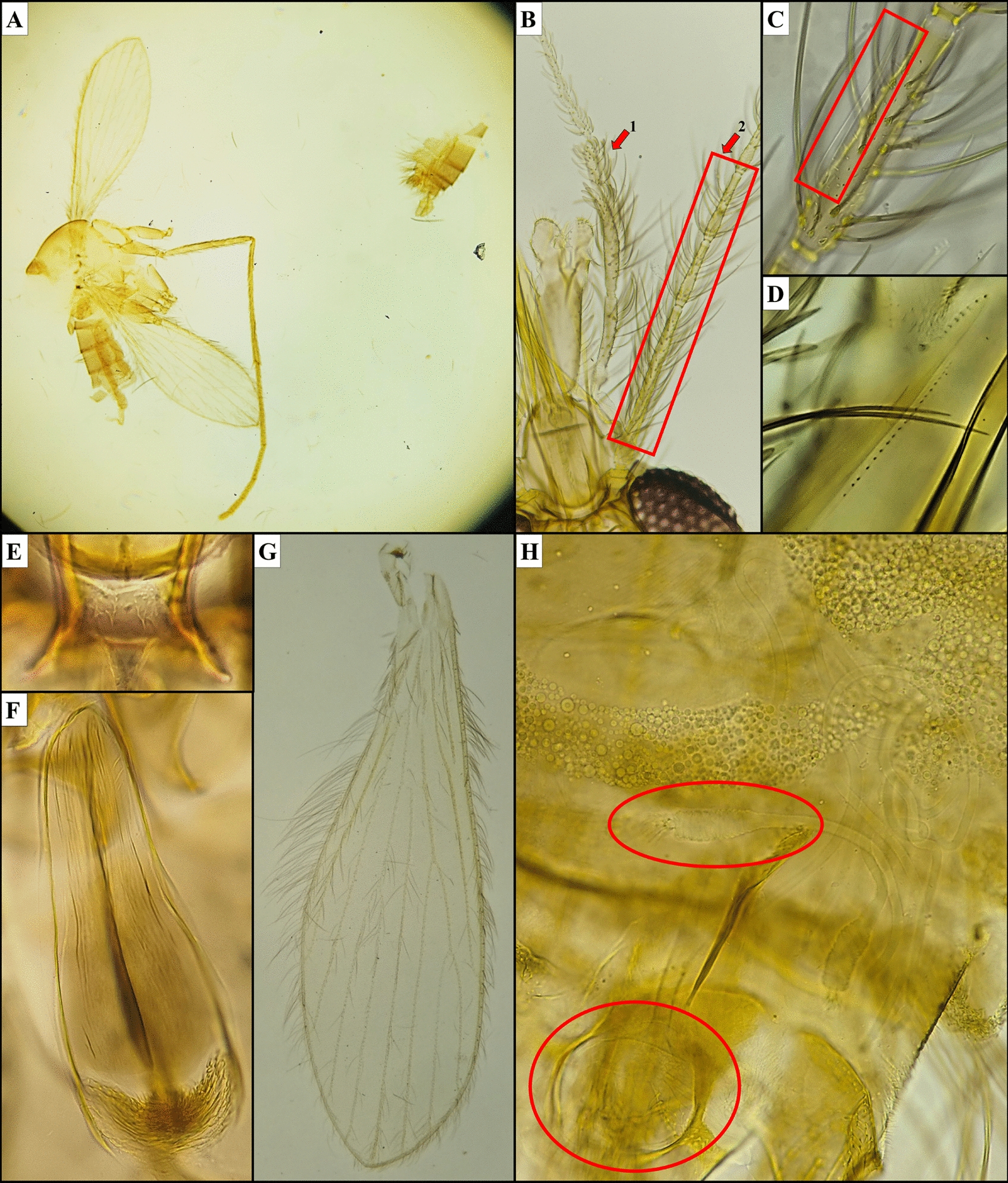
*Phlebotomus (Anaphlebotomus) ajithii* n. sp. (female). **A** Whole body without head and dissected terminalia; **B** (1) Palps, (2) flagellomere 1 to 3; **C** f2 with ascoid; **D** maxillary external and internal teeth; **E** cibarium with distinct horizontal teeth; **F** pharynx; **G** wing; **H** spermatheca with long and highly coiled individual duct

**Fig. 3:**
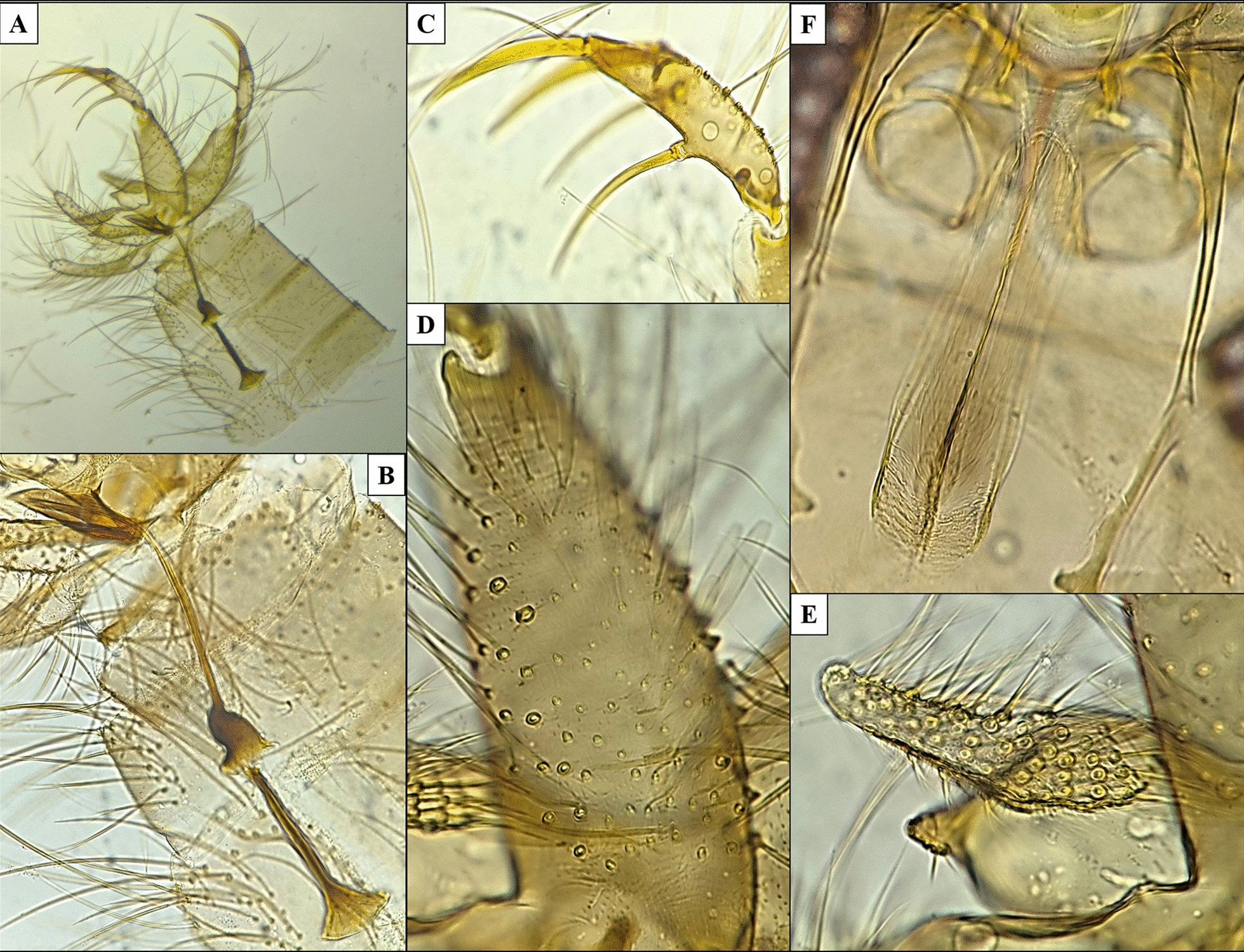
*Phlebotomus (Anaphlebotomus) ajithii* n. sp. (male). **A** Dissected terminalia; **B** sperm pump, aedeagal duct and parameral sheath; **C** gonostylus with spines arranged on it; **D** gonocoxite with internal setae on median tuft; **E** complex paramere with setae on upper lobe and a smaller ventral lobe; **F** pharynx and cibarium

Species *Phlebotomus (Anaphlebotomus) ajithii* n. sp. Shah, Fathima, Jicksy & Saini (Figs. 2 and 3).

### Female

*Holotype female*: general colour of the specimen is consistently golden brown. Body size: 1920 µm. Head: length 442 µm, width 386 µm. Interocular distance: 163 µm. Labrum is 268 µm long. Hypopharynx with about 18 teeth on each side. Number of maxillary internal teeth: 15–18; external teeth: 9–11. Palps: formula 5, 3 = 2, 1, 4 (p5 > p3 = p2 > p1 > p4). Palpomere measurements: p1 128 µm, p2 155 µm, p3 155 µm, p4 75 µm and p5 180 µm. A group of about 20 club-like Newstead’s sensilla observed on middle of third palpal segment (p3). No such structure found on other palpal segments. One distal spiniform seta observed on p3, two setae on p4 and six setae on p5. Antenna: f1 255 µm, f2 106 µm, f3 106 µm (f1 > f2 + f3). Ascoidal formula: each antenna (f1–f13) has a pair of ascoids (almost reaching the next antennal segment), one on each sides. Ascoid length in f2: 68 µm. No simple setae (SS) present on f1 to f10. Three SS on f11, eight on f12, ten on f13 and 20 on f14 were observed. Single distal papilla observed on f1 to f3. Papilla absent from f4 to f11. One papilla on f11, three on f12, five on f13 and three on f14 were observed.

*Cibarium*: the ventral area bears a few distinct horizontal teeth but no denticles or fore-teeth; pigment patch was absent on the dorsal plate of the cibarium. Cibarium bears about 4–5 varying distinct horizontal teeth. All the teeth are irregularly arranged, tapering to fine points. Pharynx: nearly slender in shape with relatively broad base, armed heavily with a small group of minute spicules, length 189 µm, width 74 µm, pharyngeal armature depth 63 µm. Pharynx is about 2.5 times as long as wide. Wings: length 1786 µm, width 567 µm. Length of principal vein sections: alpha 430 µm, beta 283 µm, gamma 232 µm, delta (R1 overlap) 102 µm, R5 length 1252 µm. Wing index (alpha/beta) 1.51.

*Fore leg*: coxa 275 µm, trochanter 79 µm, femur 763 µm, tibia 1013 µm, tarsomeres: T1 642 µm, T2 263 µm, T3 188 µm, T4 163 µm, T5 100 µm.

*Genitalia*: Spermathecae: tubular shaped, slightly narrow towards duct end with 13–15 clear distinct segmentations. Length 39 µm, width 16 µm. Apical segment of the spermatheca is not enlarged (short neck). Spermatheca with secretary cells at the distal end and narrow individual spermathecal duct with striations. The length of the individual spermathecal duct is about 15–20 times (585–780 µm) the length of the spermatheca, and the individual ducts were highly coiled. The common spermathecal duct is short, 54 µm long. Cerci is simple and 146 µm long. Genital furca is 63 µm long (Fig. 2).

### Male

*Allotype male*: same colour as the female specimen, i.e. consistently golden brown. Body size: 1733 µm. Head: length 384 µm, width 364 µm. Interocular distance 150 µm. Labrum is 183 µm long. Teeth on hypopharynx and maxilla were rudimentary. Palps: formula similar to that of female specimens, i.e. 5,3 = 2,1,4 (p5 > p3 = p2 > p1 > p4). Palpomere measurements: p1 98 µm, p2 108 µm, p3 108 µm, p4 68 µm and p5 161 µm. A group of about 15 club-like Newstead’s sensilla observed on middle of third palpal segment (p3). No such structure found on other palpal segments. One distal spiniform seta observed on p3, two setae on p4 and eight setae on p5. Antenna: f1 230 µm, f2 103 µm, f3 103 µm (f1 > f2 + f3). Ascoidal formula: each antenna (f1–f13) has a pair of ascoids (almost reaching the next antennal segment), one on each side. Ascoid length in f2: 51 µm. No SS present on f1 to f10. Four SS on f11, 10 on f12, 10 on f13 and 20 on f14 were observed. Single distal papilla observed on f1 to f3. Papilla absent from f4 to f11. One papilla on f11, three on f12, three on f13 and two on f14 were observed.

*Cibarium*: no visible horizontal teeth, denticles or fore-teeth, pigment patch was absent on the dorsal plate of the cibarium. Pharynx: nearly slender in shape with marginally broad base, comparatively lightly armed compared with female specimen, with a small group of minute spicules, length 158 µm, width 44 µm, pharyngeal armature depth 43 µm. Pharynx is about 3.6 times as long as wide.

*Wings*: length 1478 µm, width 477 µm. Length of principal vein sections: alpha 300 µm, beta 226 µm, gamma 110 µm, delta (R1 overlap) 50 µm, R5 length 927 µm. Wing index (alpha/beta): 1.33.

*Fore leg*: coxa 275 µm, trochanter 75 µm, femur 593 µm, tibia 835 µm, tarsomeres: T1 550 µm, T2 250 µm, T3 150 µm, T4 125 µm, T5 100 µm.

*Genitalia*: length of sperm pump is 204 µm, length of aedeagal duct is 175 µm, length of sperm pump + length of aedeagal duct: 378 µm, ratio of length of sperm pump/length of aedeagal duct is 1.17. Aedeagal duct is much shorter than the length of the spermathecal duct of female specimen. The aedeagal filament has no striation, i.e. it is smooth, straight, with rounded ends and overall slender throughout the duct. Ejaculatory apodeme of the male genitalia; 152 µm long. Gonocoxite of length 203 µm and with a median tuft of 48–50 internal setae. Gonostyle with a length of 150 µm and four thick spines: one apical, two subapical or subterminal and one basal with average length of 108 µm. Complex paramere with two lobes: long upper lobe with length of 148 µm and about 60 strong upward-facing setae, another shorter lobe (ventral process) without any such setae. Parameral sheath or aedeagus is thick distal end with tapering end and is finger-like in structure with a length of 102 µm. Length of epandrial lobes is 226 µm (Fig. 3).

### Diagnosis

The characteristics of uniformly erect hairs on abdominal tergites (2–6) found in the specimen are particular to the genus *Phlebotomus*. Cibarium has spicules, i.e. fine tooth-like structures, but not in a consistent row. Pigment patch is absent. Pharynx is armed with a minute group of teeth in the middle and behind it a few concentric lines. Palps extending further than antenna III (f3). Spermatheca has regular segmentation. Apical segment of the spermatheca is not enlarged, i.e. it has a short neck. Chitinous arch is well developed along with distinct horizontal teeth in the cibarium region of the head. Terminalia is short or medium sized. Paramere with or without short ventral process. Gonostyle with four long spines and coxite without basal process. These taxonomic characters confirm the addition of the species into subgenus *Anaphlebotomus* of genus *Phlebotomus*. Cibarium had a few distinct horizontal teeth in female but was rudimentary in male specimens. Maxillary teeth range between 15–18 on the internal side and 9–11 on the external side in the mouth parts of females. Ascoid on second antennal segment (f2) almost crossed the joint between f2 and f3 in both sexes. Spermatheca was tubular in shape with 13–15 clear distinct segmentations. The individual spermathecal duct was 15–20 times (585–780 µm) the length of individual spermatheca and was highly coiled. In males, gonostyle had four thick spines, one apical, two subapical or subterminal and one basal. Paramere with one ventral process and aedeagus with thick distal area and tapered at the proximal end. The aedeagal duct was short in length and was smooth and slender in shape. These features are distinctive in holotype female and allotype male *Ph. (Ana.) ajithii* n. sp. specimens.

### Variability

**Fig. 4:**
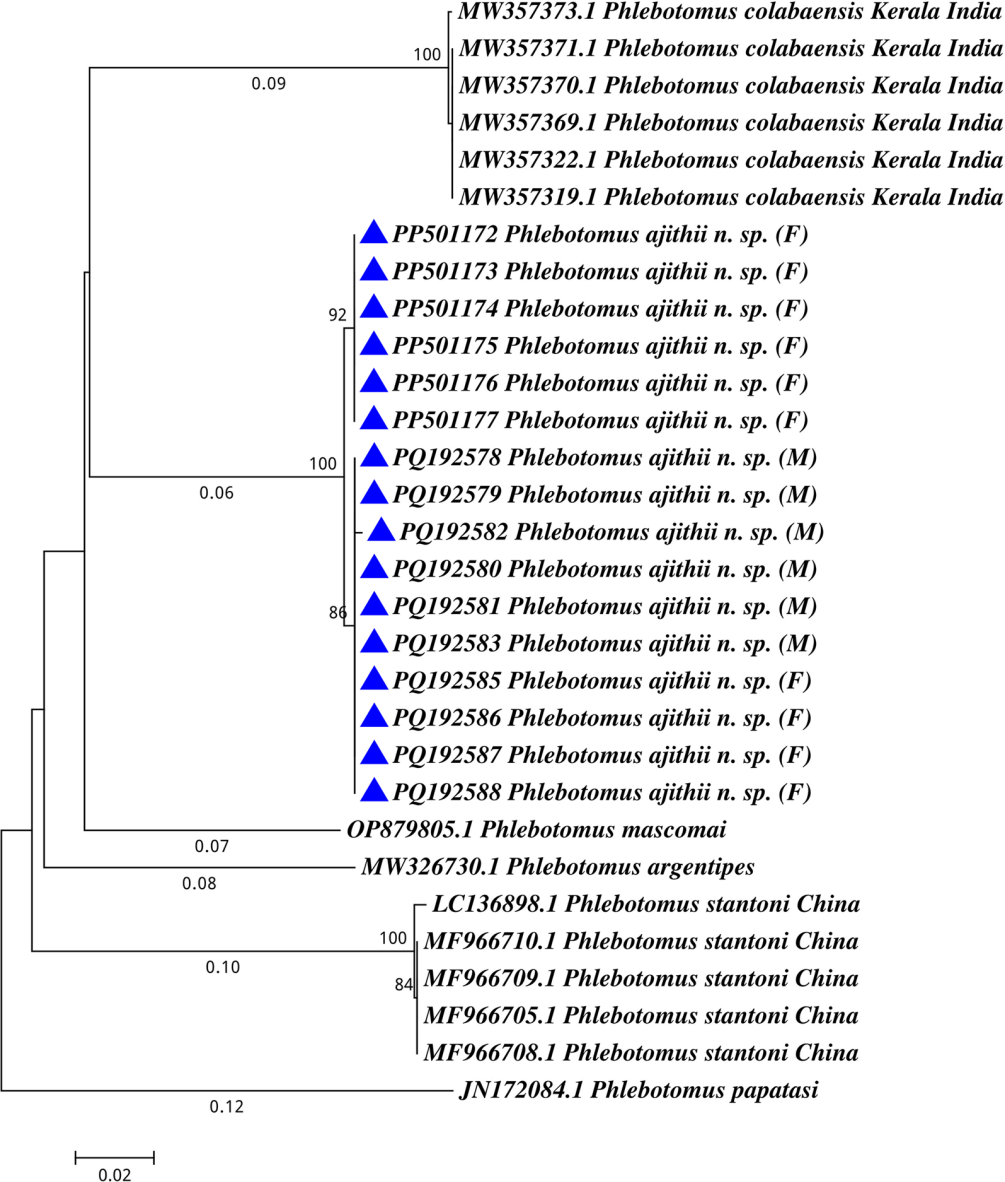
Phylogenetic tree of mitochondrial cytochrome *c* oxidase subunit I (*COI*) gene sequences for species of *Phlebotomus (Anaphlebotomus) ajithii* n. sp. along with *Ph. (Ana.) colabaensis, Ph. stantoni, Ph. (Euphlebotomus) argentipes* and *Ph. (Eup.) mascomai*; outgroup; *Ph. (Phlebotomus) papatasi*; *M* male, *F* female

The morphometric characteristics indicated that the holotype and paratype of female and allotype and paratype of males were similar (Table 3). Holotype, allotype and paratype female and male were collected from the same type of habitats but different districts. All specimens of *Ph. (Ana.) ajithii* n. sp. showed similarities in the taxonomic characteristics. In addition, DNA barcode sequences of the collected specimens from different districts showed variation in four nucleotides and an overall negligible genetic distance (K2P) within the specimens, thus suggesting a single taxonomic group. However, the genetic distance from the other most comparable congeners was 11.7% [*Ph. (Ana.) colabaensis*: 16.7%; *Ph. (Ana.) stantoni*: 17.7%] (Fig. 4).

### Type materials

*Phlebotomus (Ana.) ajithii* constitutes about 4.9% (69 specimen) of the total species composition. The type locality of *Ph. ajithii* n. sp. is cattle sheds and adjacent rooms from Kadaar tribal community of Mullurkara (Thalapilly taluk) (GPS coordinates 10.69"N, 76.25"E, altitude: 103 m above sea level). This new species has also been caught from same habitat in different districts as described in Table 1. Paratype females were obtained from the Kadaar tribe of Mullurkara tribal settlements Thrissur, Kerala, India. Paratype males were collected from Ottamala settlement, taluk-Vellarikund, Panchayat-Panathadi, District-Kasaragod, Kerala, India: (12.48"N, 75.33"E, altitude: 158 m above sea level). Voucher specimens, comprising holotype female and allotype male, were mounted separately on microscopic glass slides and serially numbered with details about the place and date of collection, habitat type, etc., and were deposited at the museum, ICMR-VCRC), Puducherry-605 006, India. Additionally, paratype female and male were subsequently submitted to the National Museum, Zoological Survey of India, Alipur, Kolkata, India.

*Phlebotomus (Ana.) ajithii* samples processed for molecular analysis were submitted to National Center for Biotechnology Information (NCBI) GenBank with accession numbers PP501172-PP501177, PQ192578-PQ192583 and PQ192585-PQ192588.

*Type specimen*: female holotype (voucher KS-265[VCRC]) and male allotype (voucher HS-1310[VCRC]) are deposited in the museum of ICMR-VCRC, Puducherry, India.

### Etymology

The new species *Ph. (Ana.) ajithii* is named after Mr. PM Ajithlal (Technical Officer ‘C’ [Retired]), ICMR-VCRC, Field Station, Kottayam, Kerala) in recognition of his unwavering commitment and lifelong dedication to the field of public health entomology.

### ZooBank registration

Following section 8.5 of the ICZN, 2012 amended version [27], details of the new species have been submitted to ZooBank. The life science identifiers (LSID) associated with the record are urn:lsid:zoobank.org:pub:FFEFC2C3-B79C-4155-A3F3-56F0AB972E0D and urn:lsid:zoobank.org:act:90BDFC54-5BCD-4168-9AAC-2A25E7F75B1A.

### Natural infection assessment of *Leishmania* parasite

None of the *Ph. (Ana.) ajithii* samples were found positive for *Leishmania* parasite.

## Discussion and conclusion

The distribution of sand flies from the rain forests of Western Ghats in India was abridged and updated by Lewis [13, 25]. The present systematic entomological survey was carried out in various tribal villages situated in the Western Ghats region of different districts in Kerala. These areas were selected because of their epidemiological relevance, i.e. case reports of leishmaniasis. These areas provide favourable macro- and microhabitat (rainfall, organic-rich soil and variety of hosts as blood sources, etc.) for the proliferation and abundance of sand flies all year. Additionally, this investigation provides data on the record of a new sand fly species from these tribal settlements.

There are several genera of sand flies, of which genus *Phlebotomus* Rondani and Berté in Rondani 1840, comprises many species from the Old World [13, 24]. Subgenera *Euphlebotomus* and *Anaphlebotomus* are the only two subgenera in which some species share common female characteristics, exhibiting pharyngeal armature, being armed and having cibarial teeth or spicules. However, females of both subgenera can be distinguished based on the apical segment of the spermatheca [13]. Species under the subgenus *Euphlebotomus* exhibit the peculiar characteristics of apical segments of the spermatheca being differentiated or enlarged compared with the others by a deeper furrow [13, 24, 30].

The subgenus *Anaphlebotomus* was one of the smallest groups under the genus *Phlebotomus*, including a total of only three species from the Oriental region [13, 24]. *Phlebotomus (Ana.) colabaensis, Ph. hoepplii* and *Ph. stantoni* are the species of the subgenus *Anaphlebotomus* which were recorded from the country [13, 24]. In 2023, this was revised to five species by Shah et al. in the review article entitled “Faunal richness and checklist of sand flies (Diptera: Psychodidae) in India” with two species records from India, i.e. *Phlebotomus (Ana.) chiyankiensis* Singh, Phillips Singh and Ipe, 2009, and *Ph. (Ana.) palamauensis Singh,* Phillips Singh and Ipe, 2007 [23]. However, as discussed by Renaux et al. in 2023, the validation of these species cannot be confirmed based only on the drawings and morphological descriptions provided by the authors [31]. Furthermore, we also accept the revision of the systematics of the subgenera *Anaphlebotomus* and reinstated the validity of *Phlebotomus maynei*, based on examination of its holotype by Renaux et al. [31]. In this continuation, a new species record, *Ph. (Ana.) ajithii* n. sp., is described in this article.

Referring to the standard taxonomic keys, the female specimens of *Ph. (Ana.) ajithii* n. sp. were found to be similar to females and males of the congener, such as *Ph. (Ana.) colabaensis, Ph. stantoni* and *Phlebotomus shadenae* [13, 24, 31]. However, based on some peculiar taxonomic characteristics, those plausible new species specimens were separated. In *Ph. (Ana.) colabaensis* female, the labrum is about 300 µm long. Hypopharynx has about 19 teeth on each side and maxilla with 11 lateral and 23 ventral teeth. Ascoid in f2 is about 60 µm. Spermatheca is slightly carrot shaped with a small end segment. Spermathecal duct is long, i.e. about four to five times length of the individual spermatheca which joins to the common duct [13, 24]. In *Ph. (Ana.) colabaensis* male, the terminalia has four spines on the gonostyle with two being apical and subapical and one basal in position. Sperm pump is 150 µm long. Gonocoxite and gonostyle are about 150 and 230 µm long, respectively. Aedeagus or parameal sheath is pyramidal in shape and sharply pointed [31]. In *Ph. (Ana.) stantoni* female, the labrum is about 230–260 µm long. Hypopharynx has about 16 teeth on each side and maxilla with nine lateral and 18 ventral teeth. Ascoid in f2 is about 80 µm. Spermatheca is fusiform or spindle shaped with about 15–16 segments; the neck is thick and short with a more or less oblong head. The spermathecal duct is slightly longer than the length of the individual spermatheca and is striated. Common duct is very long, i.e. about 1.5 times the length of the spermatheca with thick walls [13, 24]. In *Ph. (Ana.) stantoni* male, labrum is about 169 µm long. Gonostyle is 77 µm long, and sperm pump is 143 µm long. Ejaculatory apodeme is about 107 µm long. Gonocoxite is 190 µm long with 43 internal gonocoxal tuft setae. Aedeagus or parameal sheath is 88 µm long [31]. For *Ph. shadenae* female, labrum is 187 µm long. Hypopharynx has about 15 teeth on each side and maxilla with 8 lateral and 15 ventral teeth. Individual spermathecal duct is 69 µm long and has spermatheca with > 15 rings along with the presence of a sessile head carried by a broad process [31]. In *Ph. (Ana.) shadenae* male, labrum is about 167 µm long. Gonostyle is 84 µm long with four thick spines, one of which is terminal and one subterminal, and two are basal in position. Sperm pump is 139 µm long. Ejaculatory apodeme is about 107 µm long. Gonocoxite is 207 µm long with 56 internal gonocoxal tuft setae. Aedeagus or parameal sheath is 88 µm long and is finger-like in shape. Aedeagal ducts are straight, smooth and overall tapering and slender with rounded ends [31]. However, *Ph. (Ana.) ajithii* n. sp. female has about 270-µm-long labrum. The hypopharynx has about 18 teeth on each side, and the maxilla has 10 lateral and 17 ventral teeth. Ascoid present on f2 is about 68 µm. The apical segment of the spermatheca is not enlarged (short neck), which is one of the key features that differs between the two subgenera, i.e. *Euphlebotomus* and *Anaphlebotomus* [13, 24, 25]. Spermatheca is tubular and slightly narrow towards the duct end with about 13–15 segmentations. Spermathecal ducts are narrow with striations. The individual spermathecal duct is long, about 15–20 times (585–780 µm) the length of the spermatheca, and is highly coiled. It has a short common spermathecal duct of 54 µm length (Fig. 2). In the *Phlebotomus (Ana.) ajithii* n. sp. male, labrum is about 183 µm long. Ascoid length on f2 is 51 µm. Gonostyle is 150 µm long with four thick spines, one apical, two subapical or subterminal and one basal in position, with average length of 108 µm. Sperm pump is 204 µm long. Ejaculatory apodeme is about 152 µm long. Gonocoxite is 203 µm long with 48–50 internal gonocoxal tuft setae. Aedeagus or parameal sheath is 102 µm long and has a thick distal end with tapering end. Aedeagal ducts have no striation, i.e. they are its smooth and straight with rounded ends and are overall slender throughout the duct.

In addition, molecular taxonomy by DNA barcoding followed by phylogenetic analysis also confirmed the association within the specimens of *Ph. (Ana.) ajithii* n. sp. with a very minimal genetic distance and four nucleotide variations. However, the overall genetic distance (GD) is 11.7% with the congener species. *Phlebotomus (Ana.) colabaensis* has 16.7% and *Ph. (Ana.) stantoni* has 17.7% GD with *Ph. ajithii*. The population genetic parameters analysed using MEGA 7.0 software also confirmed a very high genetic diversity (*H*_*ST*_ = *0.969*) and trifling gene flow (*N*_*m*_ = *0.002*). Hence, based on these taxonomic differences and molecular analysis, *Ph. (Ana.) ajithii* n. sp. is divergent from the other already reported and described species under the subgenus *Anaphlebotomus*. This species was mainly collected from indoor human dwellings since species of the genus *Phlebotomus* are mainly incriminated in the transmission of diseases [32–36], thus, infection assessment was carried out. However, none of the specimens were positive for the *Leishmania* parasite in qPCR targeting kDNA. In conclusion, the Western Ghats is an important biodiversity hotspot with a few database on entomological surveys of sand flies. The current study tried to fill this void and also report a new sand fly species.

## Data Availability

The sequences generated in the present study were deposited in NCBI GenBank under accession nos. PP501172-PP501177, PQ192578-PQ192583 and PQ192585-PQ192588.

## Abbreviations

COI: Cytochrome *c* oxidase subunit I
NTD: Neglected tropical disease
VL: Visceral leishmaniasis
CL: Cutaneous leishmaniasis
ICZN: International Code of Zoological Nomenclature
kDNA: Kinetoplast-minicircle DNA
NCBI: National Center for Biotechnology Information
ICMR: Indian Council of Medical Research
